# Providing Care: Intrinsic Human–Machine Teams and Data

**DOI:** 10.3390/e24101369

**Published:** 2022-09-27

**Authors:** Stephen Russell, Ashwin Kumar

**Affiliations:** Department of Research, Opportunities and Innovation in Data Science, Jackson Health System, Miami, FL 33136, USA

**Keywords:** human autonomous-machine teaming, healthcare, decision support, machine learning, qualitative data, artificial intelligence

## Abstract

Despite the many successes of artificial intelligence in healthcare applications where human–machine teaming is an intrinsic characteristic of the environment, there is little work that proposes methods for adapting quantitative health data-features with human expertise insights. A method for incorporating qualitative expert perspectives in machine learning training data is proposed. The method implements an entropy-based consensus construct that minimizes the challenges of qualitative-scale data such that they can be combined with quantitative measures in a critical clinical event (CCE) vector. Specifically, the CCE vector minimizes the effects where (a) the sample size is too small, (b) the data may not be normally distributed, or (c) The data are from Likert scales, which are ordinal, so parametric statistics cannot be used. The incorporation of human perspectives in machine learning training data provides encoding of human considerations in the subsequent machine learning model. This encoding provides a basis for increasing explainability, understandability, and ultimately trust in AI-based clinical decision support system (CDSS), thereby improving human–machine teaming concerns. A discussion of applying the CCE vector in a CDSS regime and implications for machine learning are also presented.

## 1. Introduction

Like most contemporary business domains, the modern process of providing healthcare is one where there exists a complex system of interdependent human processes augmented by autonomous systems providing guidance, decision support, and in some cases even physical automation. The goal of providing ever better healthcare, along with the continuing increases in operational cost and supply chain complexities, has increased the pressure on healthcare providers to look for ways to embrace autonomous technologies. However, autonomous technologies do not necessarily mean only robots. Adopting artificial intelligence (AI), the underpinning of autonomy, in the healthcare industry has been going on for many years. Yet, healthcare continues to lag behind most other technologically driven industrial areas [1]. Using innovative AI technologies for automation has been proven to meet the increasing demands for efficient work, productivity, and managing records. Despite this lag, the adoption of autonomy in healthcare is likely inevitable, and as such, the provisioning of care will intrinsically be an environment for interdependent human–machine teams.

While there are many advancements in clinical robotic-autonomy [2,3,4,5], the broad application of autonomy as part of the business of healthcare has followed the traditional pace of the industry’s technological adoption [6]. In a recent life science executives survey, 69% of life science businesses are already piloting or have adopted AI in their solutions and 22% are evaluating or planning to pilot AI solutions [7]. The annual savings potential by using AI in healthcare has been $150 billion by 2026 in US alone, and this should be also one of the factors to speed up the implementation of AI in healthcare sector [8]. Broad adoption in an industry well-grounded in human interaction, it should not be surprising that human machine teaming should be a dominant and priority topic for researchers in this field. Many of the existing AI-based autonomous health applications have already demonstrated an imperative to involve the human stakeholders who use these systems and are the most affected by them [9]. In contrast to the situation where autonomous systems were mainly automating routine human tasks in the past, machine collaboration implies that AI systems work jointly with humans like teammates to solve problems.

Clearly, benefits can be gained by improving human machine collaboration, such as reducing labor and talent shortages through intelligent recruiting [10], lowering organizational workloads through automation [6], and improving decision support for clinical and financial outcomes [1]. However, limitations in collaborative capabilities often are codified in the data used to create the machine intelligence [11]. Underneath all AI systems are fundamental Data Science concerns and these considerations can introduce bias [12], limit trustworthiness [13], and reduce explainability [14], all critical factors for effective human–machine teams in healthcare [15]. From the perspective of healthcare professionals, a fundamental question exists about the professionals’ perception of the machine: when collaborating with AI, do they perceive AI as a teammate, or do they treat AI as a tool? For example, it is common for physicians to seek a second opinion from peers. Ideally, it should not make a difference whether the peer is another human physician or an AI system [16]. However, it is common when a physician perceives an AI system as a tool, instead of collaborative decision-making, a demand for agency becomes present when the physician overrides a system’s recommendations [17].

One reason for this is that AI often lacks qualitative context or inputs, which can moderate their objectivity and analytical functions. While objectivity is one strength of machine intelligence, it can also be a severe weakness. Particularly in automated or autonomous healthcare systems that operate as a clinical teammate, it is essential to encode human considerations as part of the AI training data. One such example of this limitation is seen in intelligent systems that employ skewed training data or training data with narrow representativeness (e.g., the under-representation of minorities) [18]. There is ample work in the literature that focus on sample bias minimization through broader or more diverse training data and other similar approaches. However, despite many calls for increased human–machine collaboration research in healthcare (e.g., [1,19,20]), there is little work in the machine learning (ML) literature that proposes methods for adapting quantitative health data-features with human expertise insights. Particularly within the AI model-training phase, approaches that allow a qualitative weighting of clinical features and that incorporate human consensus as part of training data can allow human expertise to be encoded within the machine’s intelligence. In this paper, the challenge of data and human–machine teaming in healthcare environments that adopt AI solutions is discussed. The remainder of this paper will: provide a review of previous work on human-AI collaboration in clinical settings; propose a method based on a consensus-and-dissension measure that captures human expertise as a part of the model features illustrating how healthcare professionals’ and clinicians’ input can be encoded in training data; and, finally, conclude with the practical and research considerations of integrating qualitative expertise in healthcare training data.

## 2. Background and Related Work

**Figure 1:**
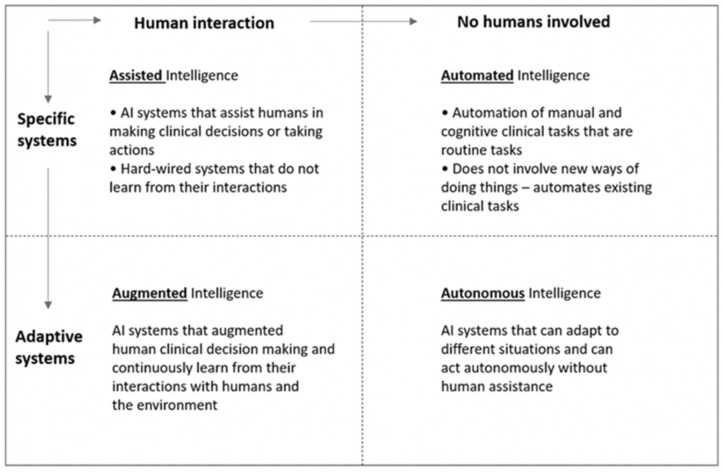
Categories of AI applications in healthcare [22].

Although the capabilities of modern healthcare AI systems have been improved with the advancement of the electronic medical record and the development of big-data deep machine learning (ML) techniques, there are still multiple challenges to achieving the benefits of seamless human machine teaming in healthcare. The increasing adoption of intelligent systems in all facets of care provisioning makes human machine teaming (HMT) an inevitable intrinsic characteristic of the healthcare environment [21]. Thus, it is critically important to address HMT concerns at multiple levels of an intelligent healthcare decision support system’s functions. Furthermore, the nature of the AI system is highly relevant as humans are always a part of the process. Even in cases where a system provides fully adaptive autonomous support, there are humans at the end points of the system’s functions. In such cases, there exists a collaborative hand-off to the human part of the HMT. Figure 1 shows categories of healthcare AI-augmented automation, illustrating how machine intelligence is applied to support human teammates.

Examining Figure 1, even for the cases where there are no humans directly involved with the machine intelligence, humans still contend with the outcomes of fully autonomous AI-driven processes. Thus, if the machine intelligence incorporates methods that create discord in expectations or engender distrust in the machine, it is detrimental to the overall support provided by the machine to the team. This characteristic is consistent with most support systems and decision support systems in particular. There is ample work in the literature that deals with HMT concerns that are largely representative of a system’s acceptance and adoption. Not surprisingly, the most common of these concerns are: validation, robustness, reliability, interpretability, transparency, and explainability [15]. The literature on these facets of system acceptance, inclusion and trust are well studied. Those concerns, represent a constant across all systems human machine teams, regardless of the category of an application in healthcare. The emphasis of this paper is on impacting the underlying intelligence and adaptive nature of systems that bring their intelligence to human–machine teams, which tend to fall in the adaptive system category.

While there is substantial research regarding the technical and engineering aspects of such intelligent systems, healthcare professionals often remain hesitant to adopt and integrate AI into their practice [23]. The rationales underneath this avoidance are still critical issues with the adoption of AI technology that implicate human-AI collaboration concerns for healthcare providers. Research has shown that over a third of healthcare professionals expressed apprehension about adopting AI due to their concerns about the alignment of an AI system’s goals with theirs, and the perceived immaturity of the technology [24]. Physicians generally avoid relinquishing entrusted patient care to a machine if it is not deemed adequately trustworthy as a teammate in clinical settings [15]. However, this reluctance is not fully rational as clinicians are surrounded by support systems, such as those that manage providers’ AI-enabled medical devices, revenue cycles, hospital operations, and resource management systems. All of these systems act as machine teammates and definitely affect, and in many cases control, critical aspects of care delivery [25]. It is these machine teammates that may present the low-hanging-fruit of opportunity for improving HMT in healthcare, but they get minimal focus in the research literature compared to clinical AI systems.

### 2.1. Artificial Intelligence, Explainability, and HMT

Of high relevance to this opportunity for improving HMT in healthcare is the prior work that has focused on AI methods and training data used to improve explainability. Researchers have pointed out many issues in using biased AI systems, e.g., diagnostic systems using datasets that are imbalanced with respect to race or other demographics [9]. Biased AI systems can diminish rather than augment human intelligence in collaborative decision teaming. Further, many ML methods, especially deep learning models, lack interpretability and transparency to their human users. They are typically “black box” models that are unable to give a rationale or an explanation for their outputs, assistance, or guidance, let alone their goals or objectives. The non-(directly) clinical systems also inherently lower many of the perceived risks typically attributed to the clinical AI teammates. Although as noted already, this lowered risk may only be perceived risks. What is definitely noteworthy is that these non-clinical AI teammates suffer from many of the underlying limitations that exist with the creation of clinical AI intelligence and training data.

The promise of explainable AI as “the answer” for HMT in healthcare has been a goal of extensive pursuits in the research literature. Interestingly, much of the literature on explainability echo the same system acceptance characteristics, validation, robustness, reliability, interpretability, transparency, etc., as are typical of any decision support system, but now aligned to ML algorithms [26]. Similarly, much of the literature also emphasizes model pairing—a interpretable “side” model that operates in conjunction with the predictive model [27] to support explainations. Another general research orientation is toward the increased use of interpretable models such as with linear regressions, logistic regressions and decision trees [28,29,30]. These ML approaches typically do not convey the power of contemporary deep learning methods (e.g., deep neural networks, deep reinforcement learning, etc.). In short, most methods that make tradeoffs in predictive and learning power are made to ensure that interpretability and explainability are maintained [30].

The approaches to achieving explainability generally do not focus on the training data in addition to trying to employ representative training data. Several prior works document explainability as essential for confidence and trust, which are critical to effective teaming and pointedly critical in healthcare [18]. There is also research that tries to achieve the necessary integration of human characteristics and machine objectivity separately or through interactive development loops, where the intelligence is iteratively refined, developed, and tested [11,30,31]. Though there is ample research, such as those noted here that target improving HMT in healthcare, few studies employ qualitative data in an integrated fashion with quantitative data to improve HMT through enriching machine intelligence training data. Research has shown that without qualitative health data, trusted healthcare AI will be imprecise, and, thus, scholars and practitioners will be missing a key component of knowledge translation efforts essential for effective HMT in healthcare applications [32]. By incorporating qualitative data into quantitative training data, human considerations can be embedded in machine intelligence, not only providing a means of explainability, but also potentially improving trustworthiness of the intelligent-machine teammate. The use of technology in a patient setting demands a complementary personal touch from clinicians. This touch is required to make certain the patient’s experience is not too clinical, rational, distant, hard-edged, cold, or impersonal [33]. 

### 2.2. Integrating Qualitative Human Perspective in Machine Learning Training-Data

Qualitative scales, such as n-point Likert scales (e.g., 5-point, 7-point, 10-point, etc.), are a commonly used means to capture human attitudes, feelings, and perspectives–essentially the degree to which a respondent reflects on an eliciting question. These scales are often given statistical treatment as if they were interval measures, inappropriately calculating means and standard deviations, and then using such distorted data in quantitative tracking and prediction models. Nonetheless, qualitative scales are simple to implement and effective in capturing human perspectives. Thus, survey scales have wide adoption in a broad variety of analytical settings Solving the underlying problems with the use, (or abuse), of qualitative scales, such as ambiguous rank-scale, improper elicitation techniques, and distributional assumptions is beyond the scope of this paper. Instead, we approach the problem from the perspective of exploiting the ability of qualitative scales to capture human perspectives, minimizing the negative effects intrinsic in their use, and applying their benefits to inform ML with human perspectives, thereby improving HMT. With that intent, some background on Likert scales, their benefits and limitations are presented here.

It is not uncommon to score “how strongly” a qualitative-factor measures on a class-based Likert or similar scale; Likert scales are widely used and accepted [34]. However, problems arise when the analysis of ordinal, categorical data is misdirected and the most frequent problem is when these measures, which are frequently numerical in nature (e.g., 1–5, 1–7, 1–10), are treated as continuous or interval data. The validity of treating scale-data as continuous or interval, parametric data, or even ratio data is uncertain. Moreover, incorrect analysis can reduce clarity and conciseness [35]. The problems caused by the initial aggregation of ordinal/categorical data are exacerbated because the results are typically used as part of further analyses (trending, classification, prediction). There is another impeding factor when these problems are taken in a ML context: the ordinal categorical data is difficult to properly integrate with other continuous and interval measures, and there is the lack of a reference, implying reference-scores to be perfection. The implied perfection reference, when combined with the issues caused by aggregation, worsens the precision problem in the non-extreme (most frequent) cases. 

Solutions have been previously proposed to address these issues, but many have significant limitations for applied ML uses. Some solutions include the use of non-parametric statistical procedures such as frequencies, tabulation, chi-squared statistics, and Kruskall-Wallis (one-way ANOVA). While effective for statistical purposes, these approaches focus on the rank or ordinal nature of the data, rather than its value. Because applying qualitative data to training data to improve HMT requires accounting for both *value and distribution*, this limitation makes such statistical aggregation techniques inappropriate. Additionally, the sparseness or unequal quantities of values can present significant challenges to many statistical methods. Other solutions emphasize changing the questions used to elicit qualitative values, i.e., re-wording survey questions. These solutions include the two-stage Likert scale [36] and phrase completion [37]. The methods adopted in these approaches require changes in the presentation and interpretation of the qualitative assessment. For example, Likert-scaled measures are typically treated as unidimensional measures of "something," while the items comprising the scale often reflect multiple dimensions. Likert-scaled elicitations are also generally worded vaguely, which results in the same item being understood differently by different respondents. These problems and others are fundamentally rooted in the format, language and technique of Likert scaling. While changing the fundamentals of Likert-scale methods would be an ideal solution, the likelihood of doing so, given Likert’s simplicity and popularity, is unlikely [38]. Rather, we accept that the use of Likert or similar measures will continue and instead, propose a method that can minimize the negative effects of their use in mixed and multi-dimensional analysis, specifically for ML methods, where they can be integrated with decision support systems and therein impact HMT concerns. 

### 2.3. Clinical Decision Support Systems, a Target for Improving HMT by Incorporating Qualitative Scales

As noted above, prior research has shown the importance of integrating human considerations in healthcare human machine teams. Qualitative data may be one way to augment training data, yet it remains infrequently used in healthcare applications beyond the behavioral and social care, and the capture of patient experiential insights [32]. By taking an approach where quantitative metrics can incorporate a qualitative and ideally, a consensus-based weight, human perspectives and explainability can be embedded in ML training data, utilized by subsequent AI systems, and ultimately improve HMT. If the underlying training data is parameterized by clinical experts, then guidance and recommendations made by such a system would likely be more palatable to their human teammates. 

The teaming of human healthcare providers with intelligent decision support systems is already pervasive in healthcare applications [39]. Clinical decision support systems (CDSS) are software that analyze data within electronic health records (EHRs) to provide prompts and reminders that assist healthcare providers in implementing evidence-based clinical guidelines at the point of care. While many think of clinical diagnosis functions as the primary domain of clinical decision support, CDSS also trigger and prioritize needs for documentation; identify and alert potential ordering conflicts and incompatibilities; and aid in provider resource utilization management. CDSS were initially designed to be used by clinicians at the point of care, but they are now being implemented for a broader range of users, essentially becoming critical team mates for all facets of care delivery [40]. Because CDSS that target clinical diagnoses have to address the elevated concerns and complexities of life and death risk, the following technical approach is focused on a CDSS that supports revenue cycle, hospital operations, and resource management.

## 3. Technical Approach

Human teaming with intelligent machines has become a fundamental characteristic of a successful CDSS. Acceptance of a CDSS for basic functions is more achievable for systems that provide simple support [22]. For more complex capabilities, including autonomous ones, adding qualitative human perspectives to machine intelligence will be important. To illustrate how qualitative factors can be incorporated in the ML training data for a CDSS, consider the following example of a documentation CDSS application. Clinical documentation is critical to the success of evidence-based care and macro-outcomes where the patient is successfully treated, administrative and operational procedures are followed, and payment is received. Because of the importance of documentation, many providers employ teams of specialized documenters who ensure that documentation is complete, accurate, and timely. These documenters have to often conduct multiple reviews, interventions, and follow-ups with clinicians as part of a patient’s treatment. From this description, it is evident that, for any provider with more than a few treatment beds, CDSS functions that provide alerting and prioritization of documenters’ work-lists are essential teaming functions.

Documentation needs to follow and support the actions and events that happen as a patient’s treatment proceeds to completion. In this paper, the treatment process hereafter will be referred to as an encounter. During the encounter there are many clinical events that occur, which signal a need for review or intervention, such as an admission without authorization; an increase in clinical consultations; surgical procedures; and other changes in treatment thresholds. In isolation, these clinical events can indicate a documentation-need but in combination with other measures, e.g., the patient is in intensive care, has co-morbidities, etc., the importance of a single measure can decline and may not warrant attention. The list of clinical events/measures/triggers may vary, but the combination of them require weighting on relative importance. It is this weight where a qualitative human perspective can be incorporated. While outside the scope of a documentation-centric CDSS, for example, there is little debate about a healthy blood pressure systolic and diastolic values, but when combined with time of day or taken as an average of *n*-measurements there exists a range of perspectives on the significance of the aggregate [41]. Similarly, in the context of a documentation CDSS, the significance of an increase in the number of consults, given an intensive care encounter, might also be weighted by a human-expert perspective. The weighting of such binary or quantitative variables is where there exists an opportunity to improve HMT concerns.

To improve a CDSS teammate, we propose a method where qualitative measures and consensuses can be incorporated to weight the quantitative ML training-data. However, such an integrated approach requires that a proper treatment be given to qualitative measures. Information theory and entropy determination can provide indications useful in quantifying the information that is contained in a sample. In this regard, entropy (a measure of the uncertainty associated with a random variable) can be useful in assessing an expected value or the average information content that is missing when a sample’s value is unknown. The approach originally proposed by Wierman and Tastle [42] adapts Shannon’s entropy (a measure of average information content) to deal with ordinal data analysis through assessing a consensus around the rank scales; thus providing a method for transforming the ordinal data into interval values, allowing them to be combined with quantitative values. This consensus approach also takes into account the data distribution, which has previously been shown to be effective in analyzing qualitative data. In this way, the use of Wierman’s and Tastle’s transformation can capture the value and variability of qualitative human inputs, conditionalize quantitative values with human perspective, and ground subsequent analyses with human-informed data.

### 3.1. Integrating Qualitative Weights as Part of a Critical Clinical Events Vector

Returning to the documentation CDSS example, quantitative critical clinical events (CCEs), such as the number of consults, the existence of surgical procedures, payer denial ratios, etc., can be constructed as a *n*-dimensional vector that represents the status of an encounter at any point in time. In a simple fashion, each dimension of the vector can be given a weight. However, the assignment of weights would need to be elicited. This elicitation can be achieved using a survey of expert opinions on the importance of the CCE variables, in context and relative to each other; e.g., given all the CCE variables, rate the importance of each variable using a Likert-scale. This rating would provide a human-perspective weight for each quantitative value. Yet, it would be statistically inappropriate to take an average of the rank-scores and then combine them with binary or parametric values due to many of the issue noted in the background section. 

To minimize the limitations of combing Likert scale values with quantitative measures, the performance consensus vector utilizes two mathematical formulations originally proposed by Weirman and Tastle [42] to obtain a consensus and its inverse (dissension). These measures provide a statistic for assessing the amount of agreement in a sample when data is extracted from a population using qualitative scales such as Likert values. The approach proposed by Weirman and Tastle finds grounding in information theory and entropy determination. Entropy, specifically the Shannon entropy [43], is useful in quantifying the information that is contained in a sample. In this regard, entropy can be useful in assessing an expected value, or the average information content that is missing when a sample value is unknown. Equation (1) shows the definition of Shannon’s entropy.

(1) $$-\sum_{i=1}^{n}p(x_{i})\log_{2}p(x_{i})$$

Weirman and Tastle adapted Shannon’s entropy to accommodate the notion of consensus and its inverse, dissention. From this perspective, Shannon entropy measures the information in a statistical distribution, particularly its dispersion. However, as seen in Equation (1), Shannon’s entropy formulation uses only the probability distribution and does not otherwise account for sample values.

(2) $$1+\sum_{i=1}^{n}p_{i}\log_{2}\left(1-\frac{\left|\;x_{i}-\mu_{x}\right|}{d_{x}}\right)$$

The consensus measure shown above in Equation (2) utilizes the sample probability distribution to determine the amount of agreement about a point on the scalar interval; in this case, the mean is represented by *µ*. Examining Equation (2), the consensus measure for the mean is defined as follows: Given a set of ranks (e.g., 5 = very important; 4 = important; 3 = neutral; 2 = marginally important; and 1 = unimportant), let *x_i_* represent a rank value from *I =* 1 to *n* (given the example provided *x*_1_ *=* 1, *x*_2_ *=* 2, and so on); *d_x_* is the width of *x_i-n_* (i.e., *maximum_x_ − minimum_x_*); *µ* is the mean of the rank-values; and *p_i_* the probability of a rank. This calculation determines the amount of sample consensus about the mean as a singular interval value between 0 and 1. 

Alone, the consensus measure can be utilized as confidence indicator of a sample’s calculated mean. Expanding this concept, Tastle and Tastle [44] extended the consensus measure by adopting a rank reference called the *strength-of-consensus*. Equation (3) shows the strength-of-consensus calculation, which is similar to the consensus measure with two exceptions. The first exception is the replacement of the mean by a rank-reference; this is shown in Equation (3) as *r_x_*. The consideration of a rank-reference is based on the notion of a preferred answer; e.g., what is the consensus if the response was 4 (important)? By assigning the mean to a specific rank-value, the consensus value is given with respect to a particular rank. A second difference between Equations (2) and (3) is seen in the division of the rank’s width (*2d_x_*). This step is necessary to keep the calculation bounded; the mathematical proof for the strength-of-consensus metric is provided in Weirman and Tastle [42], and is not included here for space considerations.

(3) $$1+\sum_{i=1}^{n}p_{i}\log_{2}\left(1-\frac{\left|\;x_{i}-r_{x}\right|}{2d_{x}}\right)$$

shows an applied example of consensus and strength-of-consensus. The example has 4 critical clinical variables (*V*_1–4_) that received 10 expert-evaluations each. Variable scores are assessed on a 1–5 rank scale: 5 = very important (V); 4 = important (I); 3 = neutral (N); 2 = marginally important (M); and 1 = unimportant (U). 

Table 1 shows the respondents’ score frequency, the mean, the standard deviation (*StDv*), consensus score (*Cns*), and each scale-rank’s strength-of-consensus. Despite each variable (*V*_1–4_*)* having the same mean (3 – neutral), their assessed importance is not the same, and it is clear that the consensus measure provides an indication of response agreement. In this way, *Cns* is similar to the standard deviation, but *Cns* is not measured in the scale domain. Strength-of-consensus indicates the degree of consensus when a specific rank is considered. 

The efficacy of Weirman and Tastle’s measure has been illustrated in several research efforts [45,46,47]. Despite the consensus measures demonstrated utility, little or no research has been conducted using it with ML techniques or in a CDSS. From an HMT and associated ML training data standpoint, the consensus measure provides several benefits. The first is the transformation of ordinal rank data into continuous interval values. Calculating consensus using base-2 *log* reduces the effects of scale disparity across experts when scoring. The distance between 2 and 3 (marginal and neutral, in the above example) or any other ranks should not be assumed the same when two different experts score an item; i.e., the difference between something being marginally important and neutral may be huge for one expert and minor for another. The log transformation applied in the consensus calculation minimizes any scale disparity effects that might exist [48]. A second benefit is gained by the nature of the consensus measure being continuous and interval; hence it is appropriate for parametric analysis techniques. A third benefit is realized in the strength-of-consensus calculation, which accounts for the assessment’s distribution and when used as a vector also capture the sample values. A fourth benefit is that both consensus and strength-of-consensus can be inverted, to provide measures of dissention.

### 3.2. Consensus Adjustment for Sample Size and Contextual Considerations

Because of the focus on agreement/consensus, Weirman’s and Tastle’s consensus metric does not account for the quantity of respondents. However, in a machine-learning context, sample size is important. Using Weirman’s and Tastle’s approach directly, it is possible to have the same consensus and strength-of-consensus values even though the two measures with different number of respondents are being compared. In this comparative sense, the consensus may be the same but 20 experts strongly agreeing is not the same as 200 experts strongly agreeing. When incorporating a consensus for each variable into a composite vector for the purposes of machine learning, it will be important to adjust the consensus value according to the number of respondents. This issue can be handled by applying a model-based weighting technique. 

For example, it is likely desirable that as the number of respondents increases, greater “weight” should be given to the consensus and strength-of-consensus. To achieve this, a sigmoid function can be applied to adjust the consensus measures in a way that progresses from a low value with an upwards slope towards a constant. Equation (4) illustrates the strength-of-consensus measure adjusted with a (Gompertz) sigmoid function, such that measures with respondent sample sizes or quantities (*q*) are logistically adjusted according to the shape of the Gompertz *S*-curve.

(4) $$\left(1+\sum_{i=1}^{n}p_{i}\log_{2}\left(1-\frac{\left|\;x_{i}-r_{x}\right|}{2d_{x}}\right)\right)\left(\;e^{-\alpha e^{-\beta q}}\right)$$

**Figure 2:**
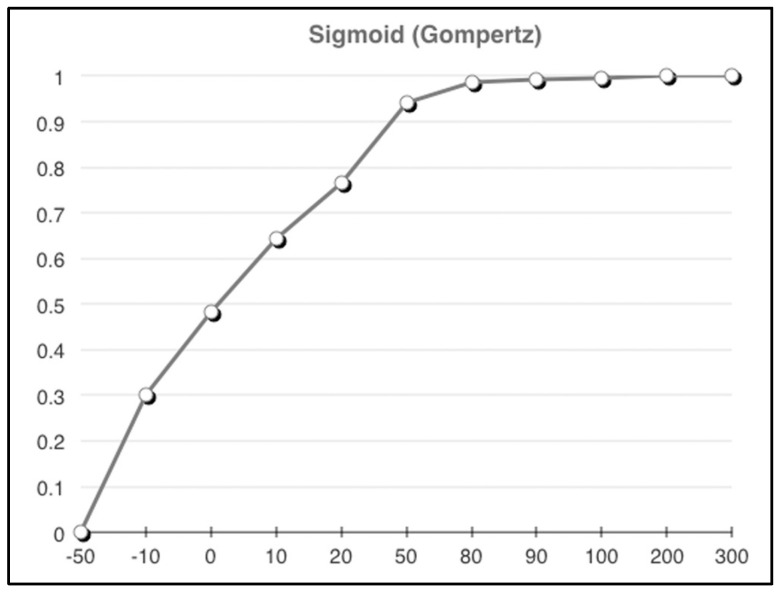
Gompertz curve weighting example.

As part of the Gompertz sigmoid, Equation (4) introduces two variables (*α* and *β*) whose values are dependent on the weighting context because they control the shape of the resulting Gompertz S-curve; *α* adjusts the minimal resulting value (the left side of the S-curve) and *β* adjusts the knee and slope of the curve. Assuming that the maximum obtainable value from the Gompertz function is 1, to produce values that adjust the weight so that quantity values above 100 are not penalized and positive values below 100 are incrementally penalized to a 50% maximum, let *α* = 0.725 and *β* = 0.05. The values of *α* and *β* change the shape of the resulting curve so that coefficients of the Gompertz-equation weight would be moderated to comply with previously mentioned penalty rules. Alternative *α* and *β* values could be selected, which would change the shape of the curve, and thus, implement other penalty values. In short, it is the shape of the Gompertz curve that provides the necessary value adjustments. Figure 2 shows an example of the resulting Gompertz curve that would be applied to provide the aforementioned weighting-adjustment to any values associated with the Gompertz equation.

**Figure 3:**
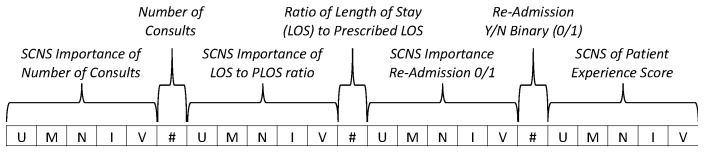
Critical Clinical Event Vector.

It should be clear from this example that a model-based weighting approach is not limited to just addressing sample size variability. Recalling that rank-scale values are actually categorical in nature and that ordering the rank numbers is somewhat arbitrary (implying that they have an equal distance between them when they do not), for some assessment purposes it is useful to have a fixed interval. If using fixed intervals each interval could be adjusted to influence the importance of one rank over another, essentially allowing training data to incorporate scale sensitivities. The model-based weighting technique can be used in conjunction with strength-of-consensus to also weight the ranks. The ability to weight the consensus transformation permits a more precise incorporation of qualitative assessment by minimizing the effects of ambiguous rank-intervals, uncertain response distributions, and sample size. The resulting consensus transformation for each qualitative measure can then be combined with other quantitative measures such as costs or counts, resulting in an *n*-dimensional critical clinical event vector like the example shown in Figure 3.

### 3.3. The CCE Vector with Integrated Consensus Scores

An example critical clinical event (CCE) vector, illustrated in Figure 3, shows a 23-dimension vector that is composed of seven metrics, each labeled by a callout. Three of the metrics (number of consults, ratio of length-of-stay (LOS) to prescribed length-of-stay (PLOS), and re-admission) are quantitative and four are qualitative (importance of number of consults, importance of LOS/PLOS ratio, importance of readmission binary, and the patient experience score). The values that comprise the vector’s qualitative-metric elements are the strength-of-consensus values for the respective rank data (very important (V); important (I); neutral (N); marginally important (M); and unimportant (U)). While Figure 3 only shows seven metrics, in practice the CCE vector may have more or fewer metrics, and it may include consensus measures depending on the application requirements.

With qualitative expert-perspective qualifiers, the CCE vector represents each encounter in a quantitative manner, such that the representation is suitable for ML training data. The consensus transformations retain explainability, while minimizing the negative effects of rank-scale elicitations. The incorporation of human-expert perspective at the lowest level of the machine intelligence provides traceability and an integration of human concerns, and becomes the basis for machine outputs and guidance. While not likely to solve the full range of HMT concerns, the CCE vector provides a construct with tunable flexibility to incorporate human concerns in machine intelligence and to improve trust in an AI-based CDSS as a teammate in healthcare applications. 

## 4. Discussion of Similarity, Machine Learning, and Human Machine Teaming in Decision Support

Two predominant hurdles have been theorized that potentially compromise a clinician’s willingness to integrate ML models into their work. First, experts may struggle to develop trust with ML-based systems due to a large number of inputs and the complex integration of data involved. The complexity of the data can make it challenging or impossible to convey the specific logic behind an alert or a recommendation [49]. Second, some evidence suggests that many view ML as too objective or too narrowly focused relative to human expertise, and they question whether it can add clinical value for highly trained expert users [50]. Use of the CCE vector in ML training data seeks to minimize these two concerns by incorporating expert perspectives in a statistically sound manner. Data constructed in the CCE vector provides a transformative basis on which ML models can be trained. However, the ML methods that are applied are highly relevant to the success of HMT with a CDSS teammate. As a reminder, the target CDSS for the CCE vector is not one making providing clinical diagnoses. Rather, the CCE vector method targets a CDSS that provides guidance on operations, administrative, or financial concerns; this section provides some discussion of the appropriate methods in which to use the CCE vector as the underlying ML training data for a CDSS.

### 4.1. Similarity—A Simple Approach to Referenced-Based Guidance

Being able to relate two things is highly relevant to human cognition and decision-making. As such, explainable similarity is a factor that should be considered in HMT contexts. Research has shown that cognitive association is a common method that aids, and in some cases hinders, decision-making–the more similar two elements are perceived to be, the more likely they are associated with the same category [51]. Additionally, referential familiarity can improve understanding in HMT contexts. Given that the CCE vector represents clinical events that can be composed at any point in an encounter, it is possible to compare encounters and relate them as a function of how similar their values are. The notion of better or worse, and good or bad, can be defined by consensus weighting relative to all of the encounters’ CCE vectors. However, this comparison is relative. A more precise, but not always feasible, approach would be to have a reference vector that defines the baseline for alerts, recommendations, or bounded-guidance. This reference CCE vector would provide a focal point in an *n*-dimensional space, such that there exists a baseline or *ideal* reference. The single, or set of, reference CCE vectors may have artificial ideal values or they may be an actual real “ideal” encounter(s). The reference values would undergo the same consensus transformation and vector composition. Given a valid reference, the vectors can be mathematically compared resulting in a similarity statistic for each vector.

By applying similarity analysis to encounter CCE vectors and a reference CCE vector, a deterministic continuous measure of performance can be acquired that will provide decision support. There are several techniques that will calculate similarity between two vectors, euclidian distance being one of the most common. Euclidian distance calculates the length of the path connecting two points in *2…n*-dimensional space. Equation (5) shows an example of the calculation for determining the distance (*d*) between two 3-dimensional points (*x*_1_, *y*_1_, *z*_1_) and (*x*_2_, *y*_2_, *z*_2_), and Equation (6) shows the generalized form of euclidian distance for points *a* and *b* with *n*-dimensions.

(5) $$d=\sqrt{{\left(x_{2}-x_{1}\right)}^{2}+{\left(y_{2}-y_{1}\right)}^{2}+{\left(z_{2}-z_{1}\right)}^{2}}$$

(6) $$d=\sqrt{\sum_{i=1}^{n}{\left(a_{i}-b_{i}\right)}^{2}}$$

Another popular distance measurement is the cosine angle distance, which measures similarity by computing the cosine of the angle between two vectors. Euclidian and cosine distances are only two of numerous distance statistics, and they have limitations that are germane to decision support. While presented here because of their common use and simplicity, both cosine and euclidian distances have significant limitations in a CDSS context. For example, both statistics assume the dimensional values are orthogonal. Both statistics also lack directional implications–they are blind to correlated or inversely correlated vectors.

Alternatively, perhaps preferentially, the Mahalanobis statistical distance (e.g., Hotelling’s T^2^ statistic) may be applied to encounter CCE vectors, as this distance calculation accounts for the directional correlations between vectors. The CCE vectors may be normalized before the distance calculation to put the vectors into the same coordinate scale, e.g., 0–1. This step will calibrate the different dimensions, recalling that appropriate weighting may have been applied during the CCE vector composition. Equation (7) shows the general form for calculating the Mahalanobis distance (*d*) between two vectors, *x* (an assessed encounter) and *y* (the reference CCE vector), where *S* is the vector’s covariance matrix.

(7) $$d(\vec{x},\vec{y})=\sqrt{{\left(\vec{x}-\vec{y}\right)}^{T}S^{-1}(\vec{x}-\vec{y})}$$

The Mahalanobis distance quantifies the dissimilarity between the ideal performance reference and the assessed entity, providing a single continuous value for each encounter’s CCE vector that indicates the encounters similarity to (distance from) the reference and each other. 

The use of similarity statistics and a reference to identify variances, trigger alerts, and supply guidance provide a relatable and understandable method on which to base decision-related support. Notions of similarity appear to play a fundamental role in human learning, and thus psychologists have done extensive research to model human similarity judgement [52]. Human judgments crucially involve determining the similarity between an object of interest and some other relevant entity and then basing the judgment on a resulting degree of similarity. Therefore, a CDSS implementing similarity statistics, would have a cognitive model that its human teammates would intrinsically understand. Moreover, similarity is a means of classification that can be applied to ranking encounters, trended against time, or utilized for other decision related analyses such as prediction, classification, or clustering. 

### 4.2. Machine Learning and Decision Support

Before machine intelligence can be deployed in a healthcare CDSS, the ML models need to be trained with data that is representative of clinical activities, such as screening, diagnosis, treatment assignment and so on. This is so the machine can learn similar groups of encounters, associations between subject features and other outcomes of interest. The CCE vector provides a construct for representative training data that also incorporates qualitative human perspectives. However, training data is only half of the ML equation; a ML model approach is also required. Despite the popularity and successes of Deep Neural Nets and other deep-learning techniques, most healthcare data is suitable for traditional machine learning methods [25]. Because of the need for categorization and recommendation, machine learning approaches such as K-nearest neighbor (KNN) and k-Means clustering are applicable and of particular interest. Both of these approaches are common, well-understood ML techniques, which also makes them suitable for CDSS applications. 

KNN and k-Means are methods for classifying or clustering entities based on their relationship to other entities. While clustering and classification may seem the same, they are different in their orientation. Classification assumes known partitions or containers, whereas clustering does not. Building a KNN using CCE vector training data can assist decision makers with taking an appropriate action, given a derived encounter class. Extending this notion further, as the underlying knowledge base is developed, the CCE vector-based CDSS can suggest guidance for corrective or lauding activities. Moreover, the application of both KNN and k-Means provides a point of departure for expert system capabilities to be integrated in the CDSS, based on the learned models.

When performance classes are not known or need to be validated, k-Means is applicable. K-Means partitions CCE vector data into *k* clusters where each encounter belongs to a cluster with the nearest mean. The k-Means method takes the number of clusters (*k*) as input and iterates to determine which encounter fits in which cluster. This type of machine learning can identify previously unknown relationships in data. For example, if encounter CCE vectors are evaluated against other characteristics new classifications may be discovered. For example, natural partitions may exist between patient satisfaction and length of stay. The identification of this relationship may allow predictions in the case of new encounter CCE vectors. To support further clustering analysis, individual dimensions of the CCE vector can be extracted and applied to k-Means. This type of analysis might reveal relationships within the CCE vector itself. Further, KNN and k-Means can be applied to encounter CCE vectors over time. This perspective can be useful for trending and prediction related analyses. Moreover, the CCE vector does not have to be limited to these machine learning approaches–other techniques may be applicable.

## 5. Conclusions

The CCE vector transformation combined with similarity or ML approaches can help to discover patterns and trends, as well as surprises in operational healthcare data. This approach allows qualitative data to be integrated with quantitative data in a manner that increases precision. When incorporated in a CDSS the CCE vector forms the basis for improved human machine teaming. However, a number of practical considerations should be considered to make the CCE vector technique more broadly useful and generalizable. Other distance calculations, such as the Canberra distance, which is sensitive to small variances from zero (i.e., for items that are very close together), may provide different benefits or greater fidelity depending on the underlying CCE vector distributions. Isolating one measure for factor analysis may be affected by weighting. The issues of sample size are related to the weighting and may emphasize undesirable effects. The CCE vector increases the computational complexity and issues of scale may arise when a dataset becomes too large. 

Despite these potential pragmatic limitations, the CCE vector addresses the challenges of adapting quantitative health data-features with human expertise insights. It does so by integrating qualitative data with quantitative data into a composite CCE vector that can be used for ML training data to allow human expertise to be encoded within machine intelligence. This encoding provides a basis for increasing explainability, understandability, and ultimately trust in an AI-based CDSS, thereby improving HMT concerns. This paper presents a CCE vector method and provides a discussion of its applications. While this presentation does not focus on experimental results, it does illustrate the CCE concept and provide details on implementation. Prior work implementing the benefits of the consensus measure has been shown in several works from the literature. As a priority, future work is planned to design a CDSS that incorporates the CCE vector method and to evaluate the integrated system in a real-world clinical HMT setting.

## Figures and Tables

**Table 1:** Example consensus results, illustrating Likert limitations.

	*Assessment Score Frequency*								*Strength-of-Consensus*				
	U (1)	M (2)	N (3)	I (4)	V (5)	Mean	StDv	Cns	U	M	N	I	V
**V1**	5	0	0	0	5	3.000	2.000	0.000	0.500	0.565	0.585	0.565	0.500
**V2**	2	2	2	2	2	3.000	1.414	0.425	0.543	0.704	0.757	0.704	0.543
**V3**	0	3	4	3	0	3.000	0.775	0.605	0.573	0.798	0.884	0.798	0.573
**V4**	0	0	10	0	0	3.000	0.000	1.000	0.585	0.807	1.000	0.807	0.585

## Data Availability

Data sharing is not applicable to this article.
